# Association of carotid atherosclerotic plaque and intima-media thickness with the monocyte to high-density lipoprotein cholesterol ratio among low-income residents of rural China: a population-based cross-sectional study

**DOI:** 10.1186/s12889-023-17447-0

**Published:** 2023-12-19

**Authors:** Zhen Zhang, Yannan Gao, Zejian Li, Bingyi Li, Shuai Gao, Jiayi Sun, Jun Tu, Xianjia Ning, Wenjuan Zhang, Jinghua Wang

**Affiliations:** 1 Department of Cardiology, Tianjin Medical University General Hospital, 154 Anshan Road, Heping District Tianjin, 300052 China; 2 School of Basic Medical Sciences, Guangxi Medical University, Nanning, Guangxi Province 530000 China; 3 Laboratory of Epidemiology, Tianjin Neurological Institute, Tianjin, 300052 China; 4 Key Laboratory of Post-Neuroinjury Neuro-repair and Regeneration in Central Nervous System, Tianjin Neurological Institute, Ministry of Education and Tianjin City, 154 Anshan Road, Tianjin, Heping District 300052 China; 5 Institute of Clinical Epidemiology & Evidence-Based Medicine, Tianjin Jizhou People’s Hospital, 18 Nanhuan Road, Jizhou District Tianjin, 301900 China

**Keywords:** Carotid Atherosclerosis, Intima-media thickness, Ratio of monocyte to high-density lipoprotein cholesterol, Epidemiology, Risk factors

## Abstract

**Background:**

The monocytes to high-density lipoprotein cholesterol ratio (MHR) has been identified as a potential biomarker for cardiovascular and cerebrovascular diseases. In this population-based cross-sectional study, we explored the relationships among carotid artery disease (CAD), including the presence of carotid atherosclerotic plaque (CAP) and carotid artery intima-media thickness (CIMT), the MHR, and related parameter changes.

**Methods:**

This cross-sectional study, Conducted from April to June 2019 in a rural area of Tianjin, involved middle-aged and elderly participants. Based on carotid ultrasound examinations, participants were divided into CAP and non-CAP groups. Logistic regression and Receiver Operating Characteristic (ROC) curve analyses were utilized to assess MHR’s predictive value for CAP. Gender-specific analyses were also performed to examine predictive variations. The relationship between CIMT and MHR was evaluated using linear regression.

**Results:**

Of the 2109 participants meeting the inclusion criteria, 51.6% were identified with CAP. Multivariate analysis revealed a significant association between MHR and CAP prevalence, (OR, 9.670; 95% CI, 2.359–39.631; *P* = 0.002), particularly in females (OR, 5.921; 95% CI, 1.823–19.231; *P* = 0.003), after adjusting for covariates. However, no significant correlation was found between CIMT and MHR when adjusted for other factors. The ROC analysis showed the area under the curve for MHR and CAP to be 0.569 (95% CI: 0.544–0.593; *P* < 0.001).

**Conclusions:**

These findings suggested that it is crucial to enhance early screening and intervention for CAD, specifically focusing on the prevention and progression of CAP, to address the unique health challenges faced by low-income groups in rural settings. Emphasizing these preventive measures could significantly contribute to improving cardiovascular health outcomes in this vulnerable population.

**Supplementary Information:**

The online version contains supplementary material available at 10.1186/s12889-023-17447-0.

## Background

Atherosclerosis is firmly established as the cause of clinical cardio-cerebrovascular disease (CCVD), a generalized term for all vascular heart and brain diseases [1]. CCVD is common, poses a serious threat to human health, and is the leading cause of death worldwide [2, 3]. Within this class of disease, cardiovascular diseases are the main cause of death in developed Western countries and in China [4]. The mortality associated with cardiovascular diseases in rural areas of China has exceeded that in urban areas since 2009, accounting for 46.74% and 44.26% of deaths in rural and urban areas, respectively [5]. The examination of atherosclerotic plaques is crucial for identifying and preventing cardio-cerebrovascular events. However, traditional ultrasound examinations are under the influences of subjective factors and anatomical variations. Thus, having a reliable and convenient laboratory marker to assess CAPs is important for clinical laboratories.

Increasing evidence indicates that the inflammatory process is closely associated with atherosclerosis [6]. The ratio of monocytes to the high-density lipoprotein cholesterol level (MHR) is a novelly proposed inflammatory and oxidative stress index that can be identified by using routine blood tests [7]. In previous studies, the MHR has been reported to be closely associated with hypertension [8], atrial fibrillation [9], coronary heart disease [10] and cerebrovascular accidents [11]. A direct relationship between carotid artery disease (CAD) and MHR has been reported in only a few populations. One retrospective study suggested that the MHR could be a marker for assessing carotid artery intima-media thickness (CIMT) in patients with systemic lupus erythematosus (SLE) [12]. In addition, a study involving 209 adult patients with acute ischemic stroke (AIS) confirmed that the MHR was an independent predictor of CAD in this population [13]. Another study suggested that the MHR is superior to traditional lipid variables for identifying CIMT increases and is an independent predictor of CIMT progression in patients with type II diabetes (T2DM) [14]. However, little is known regarding the role of the MHR in evaluating CAP and CIMT in low-income individuals living in rural China.

In this population-based cross-sectional study involving individuals who underwent ultrasound carotid wall examinations, we investigated the relationships among CAD (including CAP incidence and CIMT), MHRs, and related parameter changes, and further performed gender subgroup analysis.

## Methods

### Participants

This population-based, cross-sectional survey was implemented from April 2019 to June 2019 among middle-age and older residents of rural Tianjin, China. This population-based cross-sectional study was derived from an ongoing longitudinal cohort study program in Tianjin Brain Study. Briefly, the Tianjin Brain Study is a population-based stroke surveillance project including 14,251 participants from 18 administrative villages in the Yangjinzhuang township of Ji country in Tianjin, China [15]. This is a low income, low education level, and high incidence of stroke. The 95% local residents were farmers with relatively low levels of income. The main source of income was grain production, and the residents had an annual per capita income of <$100 in 1991 and <$1000 in 2010 [16]. According to traditional risk assessments, clinical signs and symptoms, and correlation detection, patients with the following diseases or status were excluded: cerebrovascular diseases, serious liver and kidney diseases, blood diseases, malignant tumors, and autoimmune diseases. Inclusion of population underwent ultrasonographic carotid wall examinations and were correspondingly divided into two groups according to the results: the carotid arterial atherosclerotic plaque group and the non-carotid arterial atherosclerosis plaque group.

This study was approved by the Ethics Committee of the Tianjin Medical University General Hospital and complies with the Declaration of Helsinki. All participants provided written informed consent.

### Clinical data and laboratory tests

The collected epidemiological and clinical characteristic data included participant sex; age; average annual income, lifestyle (cigarette smoking, alcohol consumption); and history of diabetes, hypertension and post-menopause. These data were collected by formally trained epidemiology investigators using a form of face-to-face interview through a predesigned, standardized questionnaire. The participants were categorized into four age groups (< 50, 50–59, 60–69 and ≥ 70 years). Annual per capita income was categorized into five groups: <2000 RMB, 2000–3999 RMB, 4000–5999 RMB, 4000–5999 RMB, or ≥ 8000 RMB. Lifestyle information included smoking status (non-smokers, occasional smokers, current smokers, and former smokers [those who had stopped smoking for at least one year]) and drinking status (non-drinkers, occasional drinkers, current drinkers, and former drinkers [those who had practiced temperance for ≥ 1 year). Cigarette smoking was defined as smoking > 1 cigarette per day for ≥ 1 year. Alcohol consumption was interpreted as drinking > 30 mL of alcohol per week for ≥ 1 year. Hypertension was defined as SBP ≥ 140 mmHg, DBP ≥ 90 mmHg, or as the existence of the requirement of medications for hypertension [17]. DM was defined as FPG level ≥ 7.0 mmol/L, a prior history of diagnosed diabetes, or requirement of insulin or oral antidiabetic drugs in the patient [18]. Post-menopause, defined by either no menstrual period in the past 12 months.

Each participant underwent a conventional physical examination, including measurement of weight, height, waistline, heart rate (HR) and blood pressure (BP). Body mass index (BMI) was computed as weight (kg) divided by the square of the height in meters (m^2^). Participants were divided into four parts according to the Chinese-specific criteria [19], including underweight (BMI < 18.50 kg/m^2^), normal (18.50 kg/m^2^ ≤ BMI < 24.00 kg/m^2^), overweight (24.00 kg/m^2^ ≤ BMI < 28.00 kg/m^2^), or obese (BMI ≥ 28.00 kg/m^2^). BP and heart rate (HR) were measured by medical professionals while the participant was in the seated position; each recorded value was the average of two measurements, collected 1 min apart, after 5 min of rest.

For routine blood and biochemical tests were drawn from all patients. Fasted venous blood samples were obtained in the morning after the participants had fasted for at least 12 h. Serum was obtained by centrifuging the sample at 3000 RPM for 10 min and then taking the supernatant. The laboratory tests included the numbers of monocytes and levels of fasting blood glucose (FBG), hemoglobin A1c (HbA1c), high-density lipoprotein cholesterol (HDL-C), low-density lipoprotein cholesterol (LDL-C), total cholesterol (TC), triglyceride (TG) and hypersensitive C-reactive protein (hsCRP), which were analyzed at Guangzhou KingMed clinical laboratory in China. White blood cell measurement was performed with an automated hematology analyser XE-1200 (KingMed, Guangzhou, China). The MHR was calculated as the monocyte count (10^9^/L) divided by HDL-C concentration (mmol/L). The MHR is divided into four groups by quartile, including Q1, Q2, Q3, and Q4.

Carotid ultrasonography was performed to evaluate CIMT and the CAP. All ultrasound examinations and measurements of participants were implemented by two trained technicians ignorant of other participant information. The participants were tested while they were in the supine position using B-mode ultrasonography instrument (t3000; Terason, Burlington, MA, USA) with a 5–12 MHz linear array transducer. The presence of a CAP was defined as an intima-media thickness greater than 1.5 mm [20, 21]. The bilateral, extracranial carotid artery systems (including the common carotid artery, carotid sinus, and internal and external carotid arteries) were screened for plaque; the size, number, shape, and location were recorded. The CIMTs of the near and far walls of the common carotid artery were bilaterally measured. The maximum, minimum, and average CIMT values on each side were recorded. The average CIMT was calculated as the mean of the sum of the bilateral CIMTs.

### Statistical analysis

Continuous variables (age, Waistline, BMI, HR, systolic BP [SBP], diastolic BP [DBP], HbA1c, FBG, TC, TG, HDL-C, LDL-C, monocyte numbers, MHR, hsCRP and CIMT) are presented as means (standard deviations) or medians (interquartile ranges). Between-group comparisons were performed using Student’s *t*-test, the Mann–Whitney test, or the Kruskal–Wallis test, as appropriate. Categorical variables (sex, age group, history of menopause, BMI group, smoking status, drinking status, annual per capita income, hypertension and diabetes) are presented as numbers and frequencies. Fisher’s exact test was used to compare categorical variables. Univariate and multivariate logistic regression analyses were applied to determine the association between CAPs and MHR, the results are presented as 95% confidence intervals (CIs); univariate and multivariate liner regression analyses were applied to determine the association between CIMT and MHR, the results are presented as β-coefficients; *P* < 0.05 was considered statistically significant. ROC curves were applied to calculate the area under the curve (AUC) of MHR and MHR quartile group. All statistical analyses were performed using the SPSS software Version 22.0 (SPSS, Chicago, IL, USA).

## Results

**Fig. 1:**
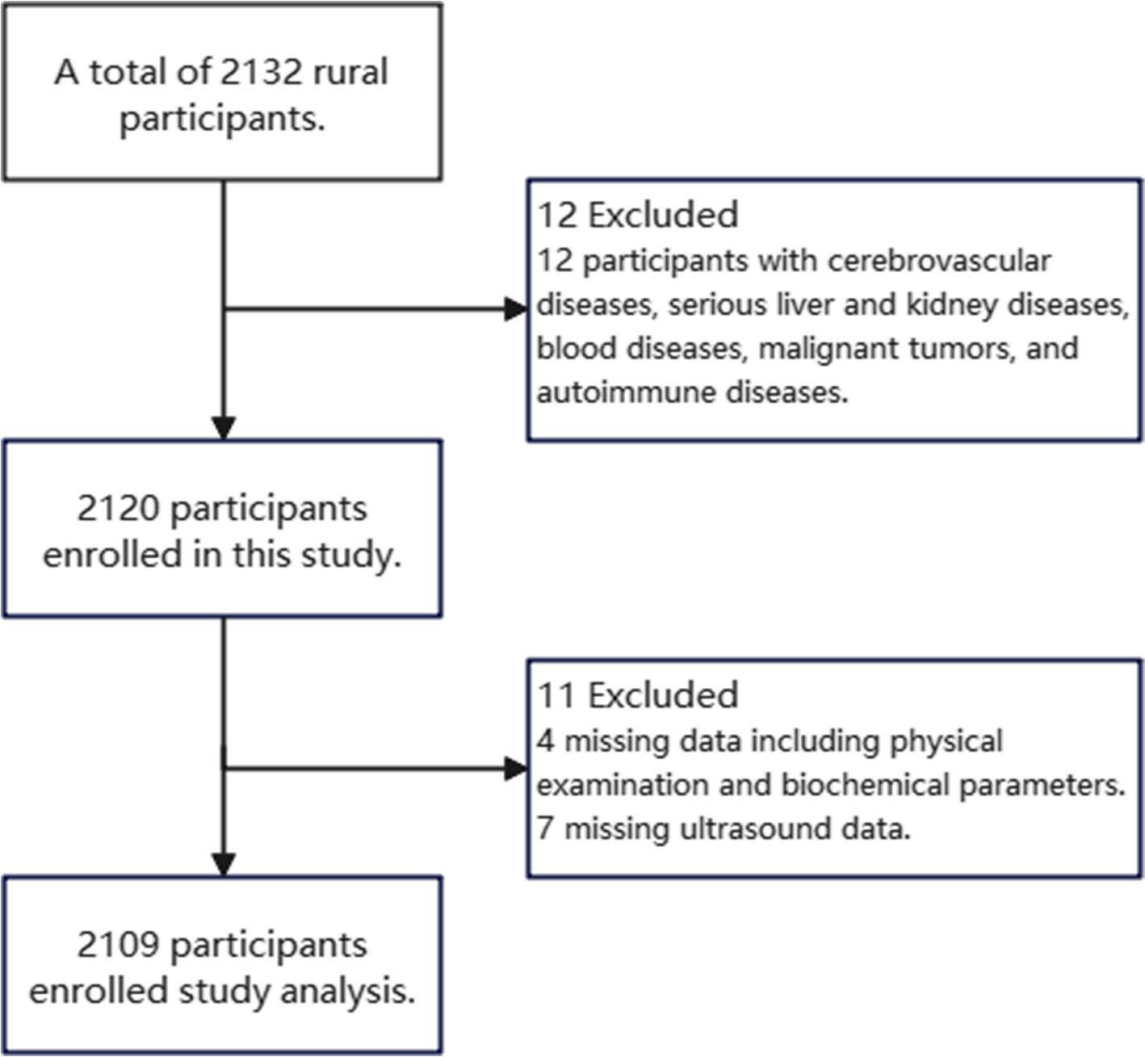
Flowchart of object selection. Of the 2132 residents who underwent the initial testing, 2120 eligible individuals enrolled in this study. Finally, 2109 participants were included in this study after excluding 11 residents with missing data, with the response rate of 98.9%

Total 2132 individuals underwent the initial testing. 2120 eligible individuals enrolled in this study. Finally, 2109 participants were included in this study after excluding 11 residents with missing data, with the response rate of 98.9% (Fig. 1).

### Baseline characteristics

Two thousand, one hundred thirty-two individuals underwent the initial testing. Based on the inclusion and exclusion criteria, 2120 eligible individuals enrolled in this study. Finally, 2109 participants were included in this study after excluding 11 residents with missing data, with the response rate of 98.9%. Of the 2109 participants, 51.6% were diagnosed with CAPs. Most of the participants were between the ages of 50 and 70 years. More than 60% of the participants had an annual per capita income of less than 2000 RMB in 2019. According to the quartile of MHR, the proportion of CAPs increased with the grade of MHR, the average value of CIMT in the highest MHR quartile group was the highest, which was 0.72 mm (Table 1).

**Table 1:** Characteristics of study participants by MHR quartile

Characteristics	MHR Quartile Group				Total
	Q1	Q2	Q3	Q4	
Cases^a^	522 (24.8)	531 (25.2)	517 (24.5)	536 (24.5)	2111 (100.0)
Age^b^, years	61.66 (8.87)	62.18 (8.82)	62.93 (8.80)	62.61 (8.69)	62.34 (8.80)
Age group^a^:					
< 50 years	30 (5.7)	35 (6.6)	23 (4.4)	25 (4.7)	113 (5.4)
50-59 years	186 (35.6)	154 (29.0)	146 (28.2)	156 (29.1)	644 (30.5)
60-69 years	213 (40.8)	244 (46.0)	240 (46.4)	244 (45.5)	943 (44.7)
≥70 years	93 (17.8)	98 (18.5)	108 (20.9)	111 (20.7)	411 (19.5)
Gender^b^:					
Men	182 (34.9)	216 (40.7)	284 (54.9)	331(61.8)	1013(48.1)
Women	340 (65.1)	315 (59.3)	233 (45.1)	205 (38.2)	1093 (51.9)
Average annual income group^a^:					
< 2000 RMB	302 (58.1)	323 (61.4)	313 (62.0)	321 (60.7)	1259 (60.5)
2000-3999 RMB	147 (28.3)	145 (27.6)	144 (27.2)	144 (27.2)	572 (27.5)
4000-5999 RMB	48 (9.2)	41 (7.8)	44 (8.3)	44 (8.3)	171 (8.2)
6000-7999 RMB	7 (1.3)	9 (1.7)	12 (2.3)	12 (2.3)	33 (1.6)
≥8000 RMB	16 (3.1)	8 (1.5)	8 (1.5)	8 (1.5)	45 (2.2)
Smoking^a^:					
Never smoking	370 (72.8)	341 (66.2)	263 (52.2)	249 (47.2)	1226 (59.5)
Occasional smoking	3 (0.6)	3 (0.6)	10 (2.0)	13 (2.5)	29 (1.4)
To give up smoking	38 (7.5)	60 (11.7)	72 (14.3)	92 (17.4)	262 (12.7)
Be smoking	97 (19.1)	111 (21.6)	159 (31.5)	174 (33.0)	543 (26.4)
Drink alcohol^a^:					
Never	304 (66.5)	294 (61.4)	282 (58.3)	265 (51.3)	1149 (59.2)
Occasional drink	38 (8.3)	52 (10.9)	60 (12.4)	85 (16.4)	236 (12.2)
To give up drink	16 (3.5)	19 (4.0)	37 (7.6)	55 (10.6)	127 (6.5)
Be drink	99 (21.7)	114 (23.8)	105 (21.7)	112 (21.7)	430 (22.1)
HR^b^, bpm	73.29 (10.96)	72.12 (11.08)	73.88 (11.92)	75.41 (13.29)	73.96 (11.90)
SBP^b^, mmHg	149.17 (20.09)	149.84 (20.02)	148.85 (20.61)	150.89 (20.42)	149.68 (20.28)
DBP^b^, mmHg	84.72 (10.78)	85.42 (11.03)	85.44 (11.30)	87.33 (11.22)	85.74 (11.12)
Hypertension^a^:					
Yes	213 (55.0)	255 (56.9)	259 (57.0)	310 (64.3)	1041 (58.6)
No	174 (45.0)	193 (43.1)	195 (43.0)	172 (35.7)	735 (41.4)
HbA1c^b^, %	6.33 (1.26)	6.59 (1.55)	6.46 (1.28)	6.39 (1.21)	6.45 (1.33)
FBG^b^, mmol/L	5.69 (1.36)	5.96 (1.81)	65.96 (1.60)	6.16 (1.79)	5.94 (1.66)
Diabetes^a^:					
Yes	47 (15.3)	64 (17.3)	73 (18.7)	83 (19.1)	267 (17.7)
No	260 (84.7)	305 (82.7)	318 (81.3)	351 (80.9)	1238 (82.3)
Waistline^b^, cm	83.62 (9.36)	86.52 (9.27)	88.05 (9.73)	90.94 (9.51)	87.30 (9.83)
BMI^b^, Kg/m^2^	24.96 (3.85)	25.92 (4.10)	25.98 (3.64)	27.25 (3.64)	26.03 (3.89)
BMI group^a^:					
Thin	14 (12.7)	3 (0.6)	5 (1.0)	0 (0.0)	22 (1.1)
Normal	216 (41.9)	159 (30.6)	142 (28.1)	88 (16.9)	606 (29.3)
Overweight	180 (35.0)	235 (45.3)	228 (45.1)	235 (45.0)	881 (42.6)
Obesity	105 (20.4)	122 (23.5)	131 (25.9)	199 (38.1)	558 (27.0)
TC^b^, mmol/L	5.18 (0.83)	5.18 (0.88)	5.10 (0.95)	5.00 (1.10)	5.12 (0.95)
TG^b^, mmol/L	1.21 (0.68)	1.43 (0.79)	1.64 (1.06)	2.24 (2.27)	1.63 (1.42)
HDL-C^b^, mmol/L	1.64 (0.36)	1.42 (0.30)	1.28 (0.27)	1.08 (0.24)	1.35 (0.36)
LDL-C^b^, mmol/L	3.11 (0.80)	3.22 (0.82)	3.16 (0.86)	3.02 (0.98)	3.13 (0.87)
MONO#^b^, 10^9/L	0.22 (0.06)	0.29 (0.06)	0.35 (0.08)	0.47 (0.21)	0.33 (0.15)
hsCRP^b^, mg/L	1.36 (3.69)	1.76 (4.43)	2.35 (4.39)	3.28 (8.34)	2.19 (5.59)
CIMT^b^, mm	0.69 (0.15)	0.69 (0.14)	0.69 (0.14)	0.72 (0.15)	0.70 (0.15)
CAP^a^:					
Yes	231 (44.3)	263 (49.6)	264 (51.1)	326 (60.8)	1088 (51.6)
No	290 (55.7)	267 (50.4)	253 (48.9)	210 (39.2)	1021 (48.4)

^a^Presented as n (%)

^b^Presented as mean (SD)

### Relationship between risk factors and the CAP (univariate analysis)

Supplemental Table 1 shows the results of the univariate logistic regression analysis used to estimate the relationship between related factors and CAPs. The prevalence of CAPs was statistical associated with both MHR and MHR quartile group (all *P* < 0.001) (Supplemental Table 1). Further, Supplemental Tables 2a and 2b detail the results from the univariate linear regression analysis, which explored the relationship between the same factors and carotid intima-media thickness (CIMT). These results indicated that CIMT has a statistically significant association with MHR (*p* = 0.005).

### Relationship between risk factors and the CAPs (multivariate analysis)

Table 2 illustrates after adjustments for gender, age, smoking status, alcohol consumption, waistline, systolic blood pressure (SBP), low-density lipoprotein cholesterol (LDL-C), fasting blood glucose (FBG), carotid intima-media thickness (CIMT), monocyte count (MONO#), and maximum heart rate (MHR), it was observed that the prevalence of CAPs is significantly associated with factors such as advanced age, smoking habits, alcohol consumption, SBP, LDL-C, CIMT, and MHR. A positive association was found between MHR and the incidence of CAPs, with an odds ratio (OR) of 9.670 and a 95% confidence interval (CI) ranging from 2.359 to 39.631 (*p* = 0.002). Supplemental Table 3 indicates no statistically significant association between CIMT and MHR after adjusting for variables including gender, age, smoking status, drinking status, waistline, SBP, LDL-C, total cholesterol (TC), FBG, and MHR.

**Table 2:** Associated factors of having carotid plaque in multivariate analysis

Factors	References	OR (95%CI)	*P*
MHR	—	9.670 (2.359, 39.631)	0.002
Gender	—	0.745 (0.521, 1.065)	0.106
Age group:	< 50 years		
50-59 years		4.112 (1.987, 8.512)	<0.001
60-69 years		7.071 (3.438, 14.541)	<0.001
≥70 years		9.064 (4.276, 19.213)	<0.001
Smoking group:	Never smoking		
Occasional smoking		0.830 (0.335, 2.055)	0.687
To give up smoking		1.547 (1.001, 2.391)	0.049
Be smoking		1.136 (0.792, 1.628)	0.488
Drink alcohol group:	Never drink		
Occasional drink		0.748 (0.506, 1.104)	0.144
To give up drink		1.878 (1.084, 3.254)	0.025
Be drinking		1.382 (0.958, 1.994)	0.084
Waistline	—	0.999 (0.988, 1.010)	0.835
SBP	—	1.012 (1.006, 1.017)	<0.001
LDL-C	—	1.261 (1.116, 1.425)	<0.001
FBG	—	1.044 (0.978, 1.115)	0.195
CIMT	—	35.42 (15.25, 82.269)	<0.001
MONO#	—	0.296 (0.069, 1.261)	0.100

### ROC curve analysis

**Fig. 2:**
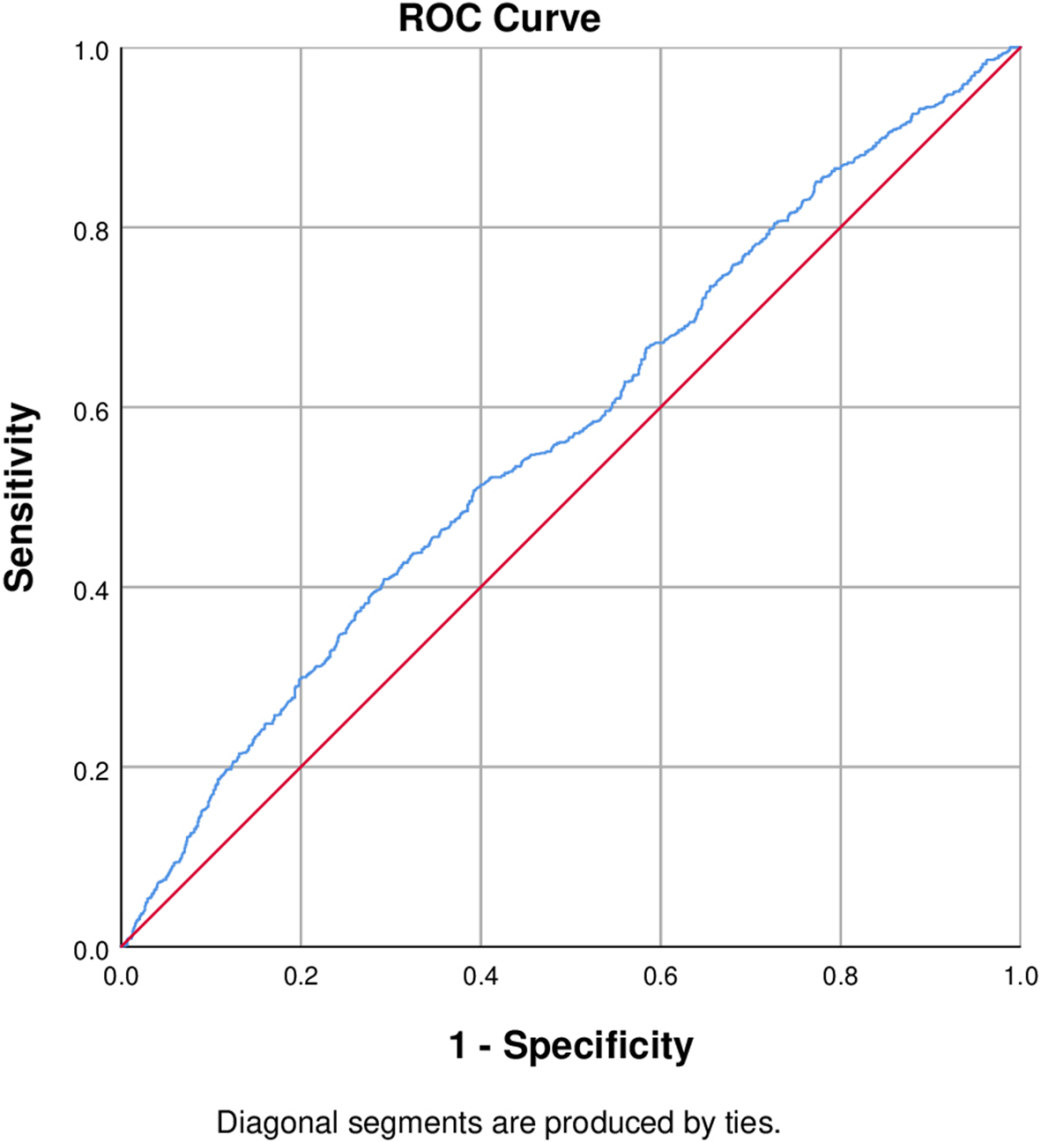
ROC curve of monocyte to high-density lipoprotein cholesterol ratio (MHR) for carotid atherosclerosis. The area under the curve was 0.569 (95% CI: 0.544-0.593; *P* < 0.001); the best threshold value is 0.285; the sensitivity is 0.409, the specificity is 0.708, and the Youden’ index is 0.117

In the ROC curve analysis assessing the diagnostic ability of MHR for CAP, the area under the curve (AUC) was modest at 0.569, with a 95% confidence interval (CI) of 0.544 to 0.593 (*P* < 0.001). Detailed analysis determined the optimal MHR threshold to be 0.285, yielding a sensitivity of 0.409 and a specificity of 0.708, with a Youden’s index of 0.117 (Fig. 2). Additionally, the ROC curve analysis for the MHR quartile group revealed a similar AUC of 0.564 (95% CI: 0.540–0.588; *P* < 0.001) (Table 3).

**Table 3:** ROC curve of diagnostic indexes of CAP

Group	AUC (95%CI)	*P*-value
MHR	0.569 (0.544-0.593)	<0.001
MHR quartile group	0.564 (0.540-0.588)	<0.001

Having carotid plaque as dependent variable

### Relationship between MHR and the CAPs by gender

Univariate logistic regression analysis showed that MHR was associated with the occurrence of plaque in the female subgroup, but not in the male subgroup (Supplemental Table 4). In multivariate analysis, age, SBP, post-menopause, and MHR were significantly associated with CAP presence in the female subgroup, after adjusting for age, post-menopause, SBP, FBG, waistline, TC, LDL-C. The OR (95% CI) was 5.921 (1.823, 19.231; *P* = 0.003) for MHR, 1.059 (1.039, 1.080; *P* < 0.001) for age, 1.017 (1.010, 1.025; *P* < 0.001) for SBP, and 3.573 (1.558, 8.194; *P* = 0.003) for post-menopause (Supplemental Table 5).

## Discussion

In the present study, we discovered a high prevalence (51.6%) of CAPs among low-income residents in one northern region of China. The prevalence of CAP was found to be associated with advanced age, give up smoking, give up alcohol, SBP, LDL-C, CIMT, and MHR, after adjusting for other covariates. Individuals with CAPs tended to have increased MHR than were non-CAP individuals. Specially, after adjusting for other covariates, MHR were significantly associated with CAP incidence in female, but not in male. Furthermore, although the AUC from ROC analysis for MHR was statistically significant (0.569, *p* < 0.001), it indicated a modest predictive value for CAP presence. This observation was consistent across MHR quartiles, which demonstrated a similar AUC of 0.564 (*p* < 0.001). These findings shed new light on the interplay between CAPs and MHR, particularly in populations from economically disadvantaged rural areas.

In China, cardiovascular diseases are the most significant cause of death and represent a grave public health problem; morbidity and mortality rates are expected to continue to rise over the next decade [22, 23]. Atherosclerosis, a systemic disease featuring to the accumulation of lipids and fibrous elements in the large arteries, is an important cause of cardiovascular disease, worldwide. A generalized review and meta-regression analysis indicated that CAPs are highly prevalent in China, especially in rural areas, and their prevalence increases with advancing age [24]. In this population-based cross-sectional study, the prevalence of CAPs was 51.6% among middle-age and older participants. Further, in line with a lot of previous studies, our study confirms that CAPs are closely age related [25].

Atherosclerosis is an inflammatory process caused by lipid accumulation in the arterial walls [26]. During the atherosclerotic process, monocytes change into foam cells and simultaneously release pro-oxidant cytokines and pro-inflammatory, leading to the collection of more monocytes and the accumulation of cholesterol ester-laden plaques. HDL-C hinders LDL-C oxidation and monocyte entry into the vascular wall, leading to endothelial or vascular protection from inflammation and oxidative stress [27]. It also inhibits the expression of monocyte tissue factors by inhibiting p38 activation and inositol phosphate kinase activity [28]. Thus, although monocytes play an active role due to their pro-oxidant and pro-inflammatory effects, HDL-C serves as a neutralizer in the process of atherosclerosis. Therefore, the relative abundance of monocytes and HDL-C (the MHR) provides an innovative indicator for assessing atherosclerosis risk by examining the association between inflammation and dyslipidemia [29].

Previous investigations have shown that MHR is an inflammatory marker in various diseases. One study suggested the association between MHR and ischemic stroke and found a linear relationship between MHR and ischemic stroke through a large cohort [29]. Some investigations have shown the function of MHR for predicting cardiovascular outcomes in metabolic syndrome and correlative atherosclerotic diseases [30–32]. However, the direct relationship between carotid artery disease and MHR has only been studied in a few populations, including patients with SLE, AIS, and T2DM. Our results showed that the MHR increased with an increase in the prevalence of carotid plaque. These results suggest that the MHR may indicate carotid plaque formation. However, more studies are needed to explore the relationship between the MHR and the occurrence and progression of carotid plaques. The MHR is an objective measurement that makes use of routine blood tests, without additional costs, providing convenience and objectivity because ultrasound examinations are significantly affected by subjective factors and anatomical variations. Thus, MHR determinations may improve the ability to diagnose CAPs. This observation is vital for the early recognition and prevention of atherosclerosis progression as well as for more detailed management policies in individuals.

The CIMT represents the measured distance between the lumen-intima interface and the media-adventitia interface of the carotid artery wall and has been suggested to be independently associated with CCVDs [33, 34]. Noninvasive ultrasonographic assessment of CIMT is applied to evaluate the early burden of atherosclerosis and predict future disease risks. In population-based studies as well as in clinical practice, the CIMT has been widely adopted as an alternative indicator for the presence of carotid atherosclerosis plaques [35]. In univariate linear regression analyses, there was a significant correlation between the MHR and CIMT in the present study. However, adjusted with other covariates, there is no statistical significance between CIMT and MHR. The further large size cohort study would be required for exploring the association of MHR with CIMT.

In this study, Individuals with CAPs tended to have increased MHR than were non-CAPs individuals, suggesting abnormal inflammation in people with CAPs. In the female subgroup, MHR was significantly associated with plaque incidence, but not in the male subgroup. The possible biological mechanism for the sex difference in the association of MHR with the incidence of CAPs is not well known. Female anatomy and specific hormone levels may be an intermediate medium to link the relationship between MHR and CAPs in female. Therefore, further research is needed to better determine the sex-differential mechanism of MHR’s association with CAP occurrence.

Our findings showed that CAP was associated with post-menopause in female. There was higher CAP prevalence in post-menopause women than that in non-menopause women. We hypothesized that it may be related to the following reasons, the hormone level changes in postmenopausal women, and the lack of estrogen protection can accelerate the progression of atherosclerosis [36]. What’s more, post-menopause is associated with a significantly higher risk of metabolic syndrome [37, 38], it includes the elevation of blood pressure, blood glucose, LDL-C and obesity [39], and the metabolic syndrome has been shown to be associated with CAPs [40].

Smoking and alcohol consumption are a major risk factor for carotid stenosis and amplifies disease severity [41, 42]. Similar to these findings, our study also showed that quitting smoking or drinking state were associated with the presence of CAPs. In this study, the prevalence of carotid plaques was correlated with SBP and LDL-C, suggesting that there were metabolism abnormalities in participants with CAPs.

### Limitations

Our study has some limitations. First, the cross-sectional design of the study prevented the observation of time-dependent changes in MHR and CAP prevalence. Second, due to the smaller sample size and regional limitations of the study, the results may not totally reflect the actual variance of the analyzed factors. Third, we did not analyze the impact of drugs used for related diseases on this result, because lower drugs’ using rate in this low-income population. Fourth, we did not enroll symptomatic patients. Finally, this study was not a clinical long-term follow-up study. Therefore, we could not objectively assess causal relationships between MHR and the development and/or progression of CAPs.

## Conclusion

Our study revealed a significant association between CAD and the MHR in low-income, middle-aged, and elderly populations in rural China, with this relationship being particularly pronounced among women. These findings suggested that it is crucial to enhance early screening and intervention for CAD, specifically focusing on the prevention and progression of CAP, to address the unique health challenges faced by low-income groups in rural settings. Emphasizing these preventive measures could significantly contribute to improving cardiovascular health outcomes in this vulnerable population.

### Supplementary Information

**Additional file 1:** **Supplemental Table 1.** Associated factors of having carotid plaque in univariate analysis. **Supplemental Table 2a.** Associated categorical variables of CIMT in univariate liner regression analysis. **Supplemental Table 2b.** Associated continuous variables of CIMT in univariate liner regression analysis. **Supplemental Table 3.** Associated factors of CIMT in multivariate liner regression analysis. **Supplemental Table 4.** Gender difference in univariate logistic regression analysis of CAP influencing factors. **Supplemental Table 5.** Gender difference in multivariate analysis of CAP influencing factors.

## Data Availability

The datasets used and/or analysed during the current study are provided by the corresponding author on reasonable request.

## Abbreviations

CCVD: Clinical cardio-cerebrovascular disease
MHR: High-density lipoprotein cholesterol level
CAD: Carotid artery disease
CAP: Carotid Atherosclerotic Plaque
CIMT: Carotid artery intima-media thickness
SLE: Systemic lupus erythematosus
AIS: Acute ischemic stroke
T2DM: Type II diabetes
BP: Blood pressure
BMI: Body mass index
HR: Heart rate
FBG: Fasting blood glucose
HbA1c: Hemoglobin A1c
HDL-C: High-density lipoprotein cholesterol
LDL-C: Low-density lipoprotein cholesterol
TC: Total cholesterol
TG: Triglyceride
hsCRP: Hypersensitive C-reactive protein
ARIC: Atherosclerosis Risk in Communities
