# Supplementary material for: Association of carotid atherosclerotic plaque and intima-media thickness with the monocyte to high-density lipoprotein cholesterol ratio among low-income residents of rural China: a population-based cross-sectional study
Source: BMC Public Health. 2023 Dec 19;23:2541. doi: 10.1186/s12889-023-17447-0 (PMC10731795; doi:10.1186/s12889-023-17447-0)
Supplement: Supplementary file 1 — Additional file 1: Supplemental Table 1. Associated factors of having carotid plaque in univariate analysis. Supplemental Table 2a. Associated categorical variables of CIMT in univariate liner regression analysis. Supplemental Table 2b. Associated continuous variables of CIMT in univariate liner regression analysis. Supplemental Table 3. Associated factors of CIMT in multivariate liner regression analysis. Supplemental Table 4. Gender difference in univariate logistic regression analysis of CAP influencing factors. Supplemental Table 5. Gender difference in multivariate analysis of CAP influencing factors. [file 12889_2023_17447_MOESM1_ESM.docx]

Supplemental Table 1. Associated factors of having carotid plaque in univariate analysis

| Characteristics | Carotid Plaque(+) Group | Carotid Plaque (-) Group | P-value |
| --- | --- | --- | --- |
| MHR^**^, % | 0.28 (0.16) | 0.25 (0.15) | <0.001 |
| MHR quartile group^*^: |  |  | <0.001 |
| Q1 | 231 (44.3) | 290 (55.7) |  |
| Q2 | 263 (49.6) | 267 (50.4) |  |
| Q3 | 264 (51.1) | 253 (48.9) |  |
| Q4 | 326 (60.8) | 210 (39.2) |  |
| Gender^*^: |  |  | <0.001 |
| Men | 622 (61.3) | 393 (38.7) |  |
| Women | 466 (42.6) | 628 (57.4) |  |
| Age^**^, years | 65.12 (7.97) | 59.39 (8.68) | <0.001 |
| Age group^*^: |  |  | <0.001 |
| < 50 years | 14 (12.4) | 99 (87.6) |  |
| 50-59 years | 228 (35.5) | 415 (64.5) |  |
| 60-69 years | 556 (59.0) | 386 (41.0) |  |
| ≥70 years | 290 (70.6) | 121 (29.4) |  |
| Smoking group^*^: |  |  | <0.001 |
| Never smoking | 546 (44.6) | 679 (55.4) |  |
| Occasional smoking | 14 (48.3) | 15 (51.7) |  |
| To give up smoking | 185 (70.6) | 77 (29.4) |  |
| Be smoking | 319 (58.9) | 223 (41.1) |  |
| Drink alcohol group^*^: |  |  | <0.001 |
| Never drink | 536 (46.6) | 613 (53.4) |  |
| Occasional drink | 119 (50.4) | 117 (49.6) |  |
| To give up drink | 95 (75.4) | 31 (24.6) |  |
| Be drink alcohol | 272 (63.3) | 158 (36.7) |  |
| HR^**^ , bpm | 73.73 (12.32) | 73.67 (11.44) | 0.916 |
| SBP^**^, mmHg | 153.54 (20.62) | 145.55 (19.08) | <0.001 |
| DBP^**^, mmHg | 86.16 (11.33) | 85.28 (10.87) | 0.076 |
| Hypertension^*^: |  |  | <0.001 |
| Yes | 636 (61.2) | 404 (38.8) |  |
| No | 336 (45.2) | 403 (54.8) |  |
| HbA1c^**^, % | 6.52 (1.37) | 6.35 (1.28) | 0.373 |
| FBG^**^, mmol/L | 6.05 (1.78) | 5.82 (1.51) | 0.002 |
| Diabetes^*^: |  |  | 0.001 |
| Yes | 168 (62.9) | 99 (37.1) |  |
| No | 636 (51.4) | 602 (48.6) |  |
| Waistline^**^, cm | 88.00 (9.76) | 86.53 (9.85) | 0.001 |
| BMI^**^, Kg/m^2^ | 25.99 (3.73) | 26.07 (4.06) | 0.640 |
| BMI group ^*^: |  |  | 0.839 |
| Thin | 10 (45.5) | 12 (54.5) |  |
| Normal | 319 (52.6) | 287 (47.4) |  |
| Overweight | 454 (51.5) | 427 (48.5) |  |
| Obesity | 281 (50.5) | 275 (49.5) |  |
| TC^**^, mmol/L | 5.19 (1.02) | 5.03 (0.86) | <0.001 |
| TG^**^, mmol/L | 1.67 (1.56) | 1.59 (1.25) | 0.199 |
| HDL-C^**^, mmol/L | 1.34 (0.36) | 1.37 (0.36) | 0.041 |
| LDL-C^**^, mmol/L | 3.21 (0.92) | 3.04 (0.81) | <0.001 |
| MONO#^**^, 10^9/L | 0.35 (0.15) | 0.32 (0.15) | <0.001 |
| hsCRP^**^, mg/L | 2.26 (4.97) | 2.11 (6.18) | <0.001 |
| CIMT^**^, mm | 0.74 (0.15) | 0.65 (0.12) | <0.001 |

^*^ Presented as n (%), ^**^ Presented as mean (SD).

Supplemental Table 2a. Associated categorical variables of CIMT in univariate liner regression analysis

| Characteristics | mean (SD) | F/t | P-value |
| --- | --- | --- | --- |
| Gender: |  | 6.853 | <0.001 |
| Men | 0.72 (0.16) |  |  |
| Women | 0.68 (0.13) |  |  |
| Age group: |  | 94.911 | <0.001 |
| < 50 years | 0.61 (0.12) |  |  |
| 50-59 years | 0.64 (0.12) |  |  |
| 60-69 years | 0.71 (0.14) |  |  |
| ≥70 years | 0.77 (0.15) |  |  |
| Smoking group: |  | 18.957 | <0.001 |
| Never smoking | 0.68 (0.14) |  |  |
| Occasional smoking | 0.72 (0.16) |  |  |
| To give up smoking | 0.75 (0.17) |  |  |
| Be smoking | 0.71 (0.15) |  |  |
| Drink alcohol group: |  | 12.885 | <0.001 |
| Never drink | 0.68 (0.13) |  |  |
| Occasional drink | 0.72 (0.16) |  |  |
| To give up drink | 0.76 (0.19) |  |  |
| Be drink alcohol | 0.71 (0.14) |  |  |
| Hypertension: |  | 5.037 | <0.001 |
| Yes | 0.72 (0.15) |  |  |
| No | 0.68 (0.13) |  |  |
| Diabetes: |  | 4.077 | <0.001 |
| Yes | 0.73 (0.14) |  |  |
| No | 0.69 (0.14) |  |  |
| BMI group: |  | 0.511 | 0.675 |
| Thin | 0.66 (0.13) |  |  |
| Normal | 0.70 (0.14) |  |  |
| Overweight | 0.70 (0.15) |  |  |
| Obesity | 0.70 (0.15) |  |  |
| Carotid plaque: |  | -15.785 | <0.001 |
| Yes | 0.74 (0.15) |  |  |
| No | 0.65 (0.12) |  |  |

Supplemental Table 2b. Associated continuous variables of CIMT in univariate liner regression analysis

| Characteristics | β (95%CI) | P-value |
| --- | --- | --- |
| MHR | 0.057 (0.017, 0.097) | 0.005 |
| Age | 0.006 (0.005, 0.007) | <0.001 |
| HR | 0.000 (-0.001, 0.000) | 0.197 |
| SBP | 0.001 (0.001, 0.002) | <0.001 |
| DBP | 0.000 (0.000, 0.001) | 0.422 |
| HbA1c | 0.001 (-0.014, 0.016) | 0.899 |
| FBG | 0.007 (0.004, 0.11) | <0.001 |
| Waistline | 0.002 (0.001, 0.002) | <0.001 |
| BMI | 0.001 (-0.001, 0.002) | 0.608 |
| TC | 0.009 (0.003, 0.016) | 0.005 |
| TG | -0.002 (-0.006, 0.003) | 0.498 |
| HDL-C | -0.003 (-0.020, 0.015) | 0.744 |
| LDL-C | 0.013 (0.006, 0.020) | <0.001 |
| MONO# | 0.061 (0.020, 0.102) | 0.004 |
| hsCRP | 0.000 (-0.002, 0.001) | 0.500 |

Supplemental Table 3. Associated factors of CIMT in multivariate liner regression analysis

| Characteristics | β(95%CI) | P-value |
| --- | --- | --- |
| MHR | 0.017 (-0.025, 0.060) | 0.423 |
| Gender | -0.021 (-0.041, -0.001) | 0.040 |
| Smoking group | 0.011 (0.004, 0.018) | 0.002 |
| Drink alcohol group | -0.005 (-0.012, 0.001) | 0.122 |
| Age | 0.005 (0.005, 0.006) | <0.001 |
| SBP | 0.001 (0.000, 0.001) | <0.001 |
| Waistline | 0.001 (0.000. 0.002) | 0.003 |
| TC | -0.007 (-0.020, 0.006) | 0.269 |
| LDL-C | 0.020 (0.006, 0.033) | 0.005 |
| FBG | 0.005 (0.001, 0.009) | 0.011 |

Adjusted OR: Adjusted with gender group, age, smoking group, drink alcohol group, waistline, SBP, LDL-C, TC, FBG and MHR.

Supplemental Table 4. Gender difference in univariate logistic regression analysis of CAP influencing factors

| Characteristics |  | Men | | | |  | Women | | | |
| --- | --- | --- | --- | --- | --- | --- | --- | --- | --- | --- |
|  |  | Carotid Plaque(+) | Carotid Plaque (-) | P-value | Total |  | Carotid Plaque(+) | Carotid Plaque (-) | P | Total |
| Age^**^, years |  | 64.49 (7.95) | 59.77 (9.09) | <0.001 | 63.27 (8.85) |  | 64.62 (7.98) | 59.14 (8.42) | <0.001 | 61.47 (8.66) |
| Age group^*^: |  |  |  | <0.001 |  |  |  |  | <0.001 |  |
| < 50 years |  | 9 (15.8) | 48 (84.2) |  | 57 (5.6) |  | 5 (8.9) | 51 (91.1) |  | 56 (5.1) |
| 50-59 years |  | 110 (46.4) | 127 (53.6) |  | 237 (23.3) |  | 118 (29.1) | 288 (70.9) |  | 407 (37.2) |
| 60-69 years |  | 327 (66.2) | 167 (33.8) |  | 495 (48.7) |  | 229 (51.1) | 219 (48.9) |  | 448 (40.9) |
| ≥70 years |  | 176 (77.5) | 51 (22.5) |  | 227 (22.3) |  | 114 (62.0) | 70 (38.0) |  | 184 (16.8) |
| Post-menopause^*^: |  |  |  |  |  |  |  |  | <0.001 |  |
| Yes |  | - | - | - | - |  | 512 (53.7) | 442 (46.3) |  | 954 (89.7) |
| No |  | - | - | - | - |  | 99 (90.0) | 11 (10.0) |  | 110 (10.3) |
| Smoking group^*^: |  |  |  | 0.001 |  |  |  |  | 0.741 |  |
| Never smoking |  | 113 (55.7) | 90 (44.3) |  | 203 (20.3) |  | 433 (42.4) | 589 (57.6) |  | 1023 (96.4) |
| Occasional smoking |  | 12 (46.2) | 14 (53.8) |  | 26 (2.6) |  | 2 (66.7) | 1 (33.3) |  | 3 (0.3) |
| To give up smoking |  | 182 (71.1) | 74 (28.9) |  | 256 (25.6) |  | 3 (50.0) | 3 (50.0) |  | 6 (0.6) |
| Be smoking |  | 305 (59.5) | 208 (40.5) |  | 514 (51.5) |  | 4 (48.3) | 15 (51.7) |  | 29 (2.7) |
| Drink alcohol group^*^: |  |  |  | <0.001 |  |  |  |  | 0.216 |  |
| Never drink |  | 142 (59.4) | 97 (40.6) |  | 239 (24.3) |  | 394 (43.3) | 516 (56.7) |  | 910 (95.1) |
| Occasional drink |  | 105 (52.0) | 97 (48.0) |  | 202 (20.5) |  | 14 (41.2) | 20 (58.8) |  | 34 (3.6) |
| To give up drink |  | 93 (76.2) | 29 (23.8) |  | 123 (12.5) |  | 2 (50.5) | 2 (50.0) |  | 4 (0.4) |
| Be drink alcohol |  | 265 (62.9) | 156 (37.1) |  | 421 (42.7) |  | 7 (77.8) | 2 (22.2) |  | 9 (0.9) |
| HR^**^ , bpm |  | 72.12 (12.39) | 72.46 (11.64) | 0.670 | 72.25 (12.10) |  | 75.88 (11.90) | 74.41 (11.27) | 0.046 | 75.01 (11.60) |
| SBP^**^, mmHg |  | 153.35 (20.93) | 147.54 (18.85) | <0.001 | 151.12 (20.33) |  | 153.79 (20.22) | 144.31 (19.13) | <0.001 | 148.34 (20.14) |
| DBP^**^, mmHg |  | 87.70 (11.27) | 89.43 (10.60) | 0.018 | 88.38 (11.05) |  | 84.09 (11.08) | 82.68 (10.22) | 0.034 | 83.28 (10.61) |
| Hypertension^*^: |  |  |  | 0.002 |  |  |  |  | <0.001 |  |
| Yes |  | 248 (68.5) | 160 (31.5) |  | 509 (59.0) |  | 288 (54.1) | 244 (45.9) |  | 532 (58.2) |
| No |  | 206 (58.4) | 147 (41.6) |  | 353 (41.0) |  | 126 (33.0) | 256 (67.0) |  | 382 (41.8) |
| HbA1c^**^, % |  | 6.46 (1.29) | 6.07 (1.02) | 0.228 | 6.35 (1.23) |  | 6.57 (1.44) | 6.45 (1.35) | 0.620 | 6.52 (1.39) |
| FBG^**^, mmol/L |  | 6.01 (1.88) | 5.76 (1.39) | 0.013 | 5.92 (1.71) |  | 6.10 (1.64) | 5.86 (1.58) | 0.019 | 5.96 (1.61) |
| Diabetes^*^: |  |  |  | 0.005 |  |  |  |  | 0.003 |  |
| Yes |  | 79 (76.0) | 25 (24.0) |  | 104 (14.7) |  | 89 (54.6) | 74 (45.4) |  | 163 (20.4) |
| No |  | 371 (61.5) | 232 (38.5) |  | 603 (85.3) |  | 265 (41.7) | 370 (58.3) |  | 635 (79.6) |
| Waistline^**^, cm |  | 88.80 (9.65) | 87.87 (9.58) | 0.134 | 88.45 (9.62) |  | 86.92 (9.81) | 85.70 (9.93) | 0.045 | 86.22 (9.89) |
| BMI^**^, Kg/m^2^ |  | 25.77 (3.57) | 25.41 (3.40) | 0.111 | 25.63 (3.51) |  | 26.29 (3.94) | 26.49 (4.37) | 0.453 | 26.41 (4.19) |
| BMI group^*^: |  |  |  | 0.799 |  |  |  |  | 0.676 |  |
| Thin |  | 6 (60.0) | 4 (40.0) |  | 10 (1.0) |  | 4 (33.3) | 8 (66.7) |  | 12 (1.1) |
| Normal |  | 190 (59.6) | 129 (40.4) |  | 319 (31.8) |  | 129 (44.9) | 158 (55.1) |  | 287 (26.9) |
| Overweight |  | 273 (61.6) | 170 (38.4) |  | 443 (44.2) |  | 181 (41.3) | 257 (58.7) |  | 438 (41.1) |
| Obesity |  | 146 (63.8) | 83 (36.2) |  | 230 (23.0) |  | 135 (41.3) | 192 (58.7) |  | 328 (30.8) |
| TC^**^, mmol/L |  | 5.02 (1.00) | 4.86 (0.80) | 0.007 | 4.95 (0.93) |  | 5.42 (1.00) | 5.14 (0.88) | <0.001 | 5.26 (0.94) |
| TG^**^, mmol/L |  | 1.58 (1.80) | 1.50 (1.54) | 0.477 | 1.55 (1.70) |  | 1.79 (1.14) | 1.65 (1.02) | 0.029 | 1.71 (1.08) |
| HDL-C^**^, mmol/L |  | 1.32 (0.37) | 1.36 (0.38) | 0.133 | 1.34 (0.38) |  | 1.36 (0.34) | 1.38 (0.35) | 0.384 | 1.37 (0.34) |
| LDL-C^**^, mmol/L |  | 3.09 (0.87) | 2.91 (0.76) | 0.001 | 3.02 (0.83) |  | 3.38 (0.96) | 3.12 (0.83) | <0.001 | 3.23 (0.89) |
| MONO#^**^, 10^9/L |  | 0.37 (0.17) | 0.36 (0.19) | 0.588 | 0.36 (0.18) |  | 0.32 (0.11) | 0.29 (0.11) | 0.001 | 0.30 (0.11) |
| hsCRP^**^, mg/L |  | 2.30 (5.93) | 2.13 (7.39) | 0.682 | 2.24 (6.53) |  | 2.21 (3.30) | 2.10 (5.29) | 0.690 | 2.15 (4.55) |
| MHR^**^, % |  | 0.30 (0.18) | 0.29 (0.19) | 0.269 | 0.30 (0.18) |  | 0.25 (0.12) | 0.23 (0.12) | 0.003 | 0.24 (0.12) |
| CIMT^**^, mm |  | 0.76 (0.16) | 0.66 (0.13) | <0.001 | 0.72 (0.16) |  | 0.73 (0.14) | 0.64 (0.12) | <0.001 | 0.68 (0.13) |

^*^ Presented as n (%), ^**^ Presented as mean (SD).

Supplemental Table 5. Gender difference in multivariate analysis of CAP influencing factors

| Factors | References | OR (95%CI) | P-value |
| --- | --- | --- | --- |
| Smoking group: | Never |  |  |
| Occasional smoking |  | 0.750 (0.291, 1.931) | 0.551 |
| To give up smoking |  | 1.623 (1.018, 2.587) | 0.042 |
| Be smoking |  | 1.244 (0.834, 1.858) | 0.285 |
| Drink alcohol: | Never |  |  |
| Occasional drink |  | 0.654 (0.422, 1.015) | 0.058 |
| To give up drink |  | 1.741 (0.990, 3.063) | 0.054 |
| Be drink |  | 1.165 (0.790, 1.716) | 0.441 |
| Age | — | 1.063 (1.043, 1.084) | <0.001 |
| SBP | — | 1.008 (1.000, 1.015) | 0.051 |
| FBG | — | 1.100 (0.996, 1.214) | 0.061 |
| TC | — | 1.030 (0.779,1.363) | 0.835 |
| LDL-C | — | 0.257 (1.191, 0.881) | 0.257 |
| CIMT | — | 26.041 (8.466, 80.104) | <0.001 |
| Women: | | | |
| MHR | — | 5.921 (1.823. 19.231) | 0.003 |
| Age | — | 1.059 (1.039, 1.080) | <0.001 |
| SBP | — | 1.017 (1.010, 1.025) | <0.001 |
| FBG | — | 1.020 (0.938, 1.108) | 0.648 |
| TC | — | 1.213 (0.880, 1.671) | 0.239 |
| LDL-C | — | 1.081 (0.772, 1.515) | 0.651 |
| Post-menopause | Non-menopause | 3.573 (1.558, 8.194) | 0.003 |
| Waistline | — | 0.989 (0.975, 1.003) | 0.128 |
